# Dual character of surface engineering on SN38 prodrug nano-assemblies: divergent effects on in vitro and in vivo behavior

**DOI:** 10.1186/s40779-025-00648-6

**Published:** 2025-09-22

**Authors:** Ya-Qiao Li, Zhi-Yu Kuang, Bao-Yuan Zhang, Yan-Zhong Hao, Ling-Xiao Li, Jing-Xuan Zhang, Ya-Fan Xiao, Bo-Wen Zhang, Xian-Bao Shi, Xiao-Hui Pu, Zhong-Gui He, Bing-Jun Sun

**Affiliations:** 1 Department of Pharmaceutics, Wuya College of Innovation, Shenyang Pharmaceutical University, Shenyang, 110016 China; 2 Department of Bioengineering, Graduate School of Engineering, the University of Tokyo, Tokyo, 113-0033 Japan; 3 Department of Pharmacy, the First Affiliated Hospital of Jinzhou Medical University, Jinzhou, 121001 Liaoning China; 4 State Key Laboratory of Antiviral Drugs, School of Pharmacy, Henan University, Kaifeng, 475004 Henan China; 5 Joint International Research Laboratory of Intelligent Drug Delivery Systems, Ministry of Education, Shenyang, 110016 China

**Keywords:** Surface engineering, Prodrug, Polyethylene glycol (PEG), SN38, Long circulation

## Abstract

**Background:**

Surface engineering has emerged as a promising strategy to enhance the performance of nanomedicines. In particular, the PEGylation levels for chemotherapy drug 7-Ethyl-10-hydroxycamptothecin (SN38) prodrug nanoparticles (NPs) play a crucial role in determining their stability, drug release kinetics, cytotoxicity, cellular uptake, in vivo pharmacokinetics, biodistribution, and antitumor efficacy. The study aims to investigate the surface engineering for chemotherapy drugs, providing new solutions for improving their in vivo delivery.

**Methods:**

We systematically evaluated the effects of different PEGylation levels on NPs (W_DSPE-mPEG2k_/W_prodrug_; 0%, 5%, 20%, 40%, 60%, 80%, 100%, 150%, and 200% NPs) incorporated on SN38 prodrug NPs via surface engineering. Drug release was measured using high-performance liquid chromatography (HPLC), while cytotoxicity was assessed via the 3-(4,5-dimethylthiazol-2-yl)-2,5-diphenyltetrazolium bromide (MTT) assay. Cellular uptake was accurately quantified using liquid chromatography–mass spectrometry (LC–MS). The in vivo pharmacokinetics of the NPs were evaluated in Sprague–Dawley rats, and the biodistribution and antitumor efficacy were assessed using a CT26 colon tumor-bearing BALB/c mice model. Additionally, we examined intestinal toxicity to evaluate the safety profile.

**Results:**

All the different PEGylation levels of SN38 prodrug NPs exhibited high drug loading (> 25%) but distinct behaviors depending on the PEGylation level. Low PEGylation (20%) led to poor colloidal stability, reduced cellular uptake, and rapid clearance by the mononuclear phagocyte system (MPS), resulting in unfavorable pharmacokinetics. Moderate PEGylation (80%) improved in vitro stability and uptake but remained insufficient to prevent rapid clearance in vivo. In contrast, high PEGylation (150%) significantly enhanced pharmacokinetic profiles, prolonged circulation, and increased tumor accumulation. The 150% NPs also showed superior antitumor efficacy without triggering anti-polyethylene glycol (PEG) immune responses or accelerated blood clearance (ABC) effects. Although high PEGylation slightly reduced cellular uptake, it conferred essential stability for systemic delivery, improving in vivo therapeutic outcomes.

**Conclusions:**

The high PEGylation (150% NPs) exhibited the best antitumor effect and the lowest degree of intestinal toxicity. Our findings underscore the critical impact of PEGylation level on enhancing the performance and safety of SN38 prodrug NPs.

**Supplementary Information:**

The online version contains supplementary material available at 10.1186/s40779-025-00648-6.

## Background

Irinotecan (CPT-11) is a widely used chemotherapeutic agent for treating colon and pancreatic cancers [1, 2]. However, its therapeutic efficacy is Limited by its inefficient conversion into its active metabolite, 7-Ethyl-10-hydroxycamptothecin (SN38) [3–5]. Conversion rates range from 0.1 to 1%, with significant variability among patients [6]. The primary challenge with the conversion of CPT-11 to SN38 at the tumor site is due to the lack of carboxylesterase expression [7]. Instead, this conversion predominantly occurs in the liver, where SN38 is further metabolized by uridine diphosphate-glucuronosyltransferase 1A1 (UGT1A1) into SN38-glucuronide (SN38G) [8], a water-soluble inactive metabolite [9]. Both SN38 and SN38G are excreted into the bile and transported to the intestine via ATP-activated transporters. In the intestine, bacterial β-glucuronidase can convert SN38G back into SN38 [10], which leads to severe intestinal toxicity [11]. Consequently, the direct delivery of SN38 has been explored as a promising strategy [12]. However, the poor solubility of SN38 in water and commonly used organic solvents poses significant challenges [13, 14].

The rational design has emerged as an effective strategy for delivering this poorly soluble drug. Compared with active-targeting antibody–drug conjugates (ADCs) such as Sacituzumab govitecan, passive targeting remains a popular nano-delivery method approved by the Food and Drug Administration (FDA) due to its simpler preparation process [15]. By linking two drug molecules with specific chemical linkers, the developed dimeric prodrugs could function as both the carrier and cargo, offering self-assembly capability and ultrahigh drug loading capacity for delivering SN38 [16–18]. In our previous study [19], we developed a series of SN38 dimeric prodrugs featuring varying linker lengths (C5, C8, C10, C12, C14, C16, and C18). Among these prodrugs, the C12-bridged prodrug achieved the optimal balance between self-assembly stability and activation efficiency, resulting in the best antitumor effects. Nevertheless, its pharmacokinetic properties were far from satisfactory, suggesting that prodrug optimization alone is insufficient for efficient SN38 delivery [20]. Surface engineering of nanoformulations offers a promising strategy to overcome these challenges. By modifying the surface, nanoformulations can evade recognition by the mononuclear phagocyte system (MPS) [21]. In particular, PEGylation has emerged as a key strategy for improving the circulation time and tumor accumulation of nanoformulations [22].

PEGylation plays a crucial role in nanoparticles (NPs) by forming a hydrophilic layer on the surface [16, 17, 23–27]. This layer prevents the aggregation and precipitation of hydrophobic small-molecule prodrugs in aqueous environments [18, 28, 29]. Additionally, adequate PEGylation reduces recognition and clearance by the MPS, thereby prolonging systemic circulation. However, an excess of PEG limits the self-assembly stability and cellular uptake of NPs [30, 31]. Thus, PEGylation levels significantly influence the in vitro and in vivo behaviors of NPs. N-(carbonyl-methoxypolyethyleneglycol-2000)-1,2-distearoyl-sn-glycerol-3-phosphoethanolamine (DSPE-mPEG_2k_), which is commonly used in commercial nanoformulations such as Doxil® [32], has proven effective in extending the systemic circulation of nanomedicines.

In this study, we chose DSPE-mPEG_2k_ as a surface engineering material to elucidate the relationship between PEGylation level and NPs behavior both in vitro and in vivo, thereby guiding rational surface engineering strategies. We designed a series of C12-bridged SN38 prodrug NPs with varying PEGylation levels (W_DSPE-mPEG2k_/W_prodrug_; 0%, 5%, 20%, 40%, 60%, 80%, 100%, 150%, and 200% NPs) using a one-step nanoprecipitation method. A LC–MS method was employed for accurate quantitative analysis. These findings may offer valuable insights for the development of long-circulating NPs for chemotherapy.

## Methods

### Characterization and stability of the SN38 prodrug NPs

The hydrodynamic diameters and zeta potentials of the SN38 prodrug NPs were determined with a Zetasizer instrument (Nano ZS, Malvern Co., UK). The morphology of the SN38 prodrug NPs was observed by transmission electron microscopy (TEM; Hitachi, HT7700, Japan).

The stability of the SN38 prodrug NPs at 2 concentrations was detected. Phosphate buffer saline (PBS) supplemented with 30% fetal bovine serum (FBS) was used to investigate the change in size of the SN38 prodrug NPs. The hydrodynamic diameter of SN38 prodrug NPs was measured immediately after FBS addition (defined as 0 h). Particle sizes were then recorded at 0, 2, 4, 6, 8, 12, and 24 h during FBS incubation. Relative particle size was calculated as the percentage of each time point diameter relative to the 0 h value, indicating the change in size over time. The stability of the SN38 prodrug NPs after centrifugal force (4000 rpm for 2 min) to disrupt the SN38 prodrug NPs to investigate their stability. HPLC was used to detect the remaining SN38 prodrug NPs. The chromatographic column used was COSMOSIL 5C18-PAQ (250 mm × 4.6 mm, 5 μm). Additionally, the flow rate was 1 ml/min, and the mobile phase was composed of acetonitrile and deionized water. The detection wavelength was 360 nm. The changes in hydrodynamic diameters were also detected.

Molecular simulations were performed using the Yinfo Cloud Computing Platform (Guangzhou Yinfo Information Technology Co., Ltd., China). The calculation parameters were as follows: the ligand molecule and receptor molecule were the prodrug and SN38, respectively; the pocket range of the prodrug and SN38 was 20; the box edge of the prodrug and SN38 was 16; the calculation mode was flexible Ligand Linkage; and the maximum number of output conformations was 9.

### Cell culture and treatment

Mouse colon cells (CT26 cells) and mouse breast cancer cells (4T1 cells) were obtained from the Cell Bank of Type Culture Collection of the Chinese Academy of Sciences (Beijing, China). CT26 cells were cultured in Dulbecco’s modified Eagle medium (Meilun Biotechnology Co., Ltd., Dalian, China). 4T1 cells were cultured in Roswell Park Memorial Institute 1640 (Meilun Biotechnology Co., Ltd., Dalian, China). Both types of media were supplemented with 1% solution of penicillin and streptomycin to ensure the prevention of bacterial contamination. Both types of media also contained 10% FBS, providing essential nutrients and growth factors necessary for optimal cell growth and maintenance. Both cell Lines were incubated in a humidified environment at a temperature of 37 °C, with a consistent concentration of 5% CO_2_, which is crucial for sustaining the physiological conditions required for cellular proliferation and viability. Drug release, cytotoxicity, cellular uptake, and intracellular release studies were conducted according to the methods described in Additional file 1: Methods.

### Animals

Ninety 10-week-old male BALB/c mice at a Weight of 20–22 g (*n* = 90) and 20 10-week-old male Sprague–Dawley rats at a Weight of 200–220 g (*n* = 20) were purchased from Liaoning Changsheng Biotechnology Co., Ltd. (Liaoning, China). The mice were maintained in a specific pathogen-free animal facility in a standard humidity and temperature-controlled environment under a 12 h/12 h light/dark cycle, with free access to food and water. All procedures were approved by the Institutional Animal Ethical Care Committee (IAECC) of Shenyang Pharmaceutical University (SYPU-IACUC-C2021-12-31-101) and complied with the National Institutes of Health Guidelines for the Care and Use of Laboratory Animals. BALB/c mice were allocated as follows: biodistribution experiment (*n* = 45), CD31 expression analysis in tumor and normal tissues (*n* = 6), in vivo anti-tumor efficacy evaluation (*n* = 30), blood routine analysis in the normal group (*n* = 3), and assessment of the accelerated blood clearance (ABC) effect (*n* = 6). Sprague–Dawley rats were used for the pharmacokinetic study (*n* = 20; 4 rats per group, 5 groups in total).

### Pharmacokinetic study

The pharmacokinetic profiles were studied in Sprague–Dawley rats. The Sprague–Dawley rats were randomly divided into 5 groups (*n* = 4), including SN38 solution (SN38 sol), CPT-11, or SN38 prodrug NPs (20%, 80%, or 150% NPs). All formulations were administered via injection at an equivalent SN38 concentration of 4 mg/kg. Since DMSO is not suitable for in vivo injection, SN38 was dissolved in a fat emulsion at 1 mg/ml for animal studies. Plasma samples were collected at predetermined times (0.033, 0.083, 0.25, 0.5, 1, 2, 4, 8, 12, and 24 h). The amounts of SN38, CPT-11, and the SN38 prodrug in the plasma were detected by LC–MS-8060. The method of LC–MS-8060 was described in Additional file 1: Methods. The pharmacokinetic properties were calculated by DAS 2.1.1 (China Drug Evaluation Network, China).

### Biodistribution study

We established tumor models by injecting CT26 under the skin on the backs of BALB/c mice (*n* = 45). When the tumor volume reached 300 mm^3^, the mice were divided randomly into 5 groups and received SN38 sol, CPT-11, or SN38 prodrug NPs [20%, 80%, and 150% NPs (at an equivalent SN38 concentration of 4 mg/kg); *n* = 3 in each group at each time point]. The mice were subsequently euthanized 1, 4, or 12 h after administration. Then, the organs and tumors were collected, and the levels of the SN38, CPT-11, and SN38 prodrug NPs in the heart, liver, spleen, lung, kidney, and tumor were quantitatively analyzed via LC–MS-8060. The method of LC–MS-8060 was described in Additional file 1: Methods. The vascular distribution and density were assessed using platelet endothelial cell adhesion molecule-1 (CD31) antibody (Servicebio, Wuhan, China). The normal tissues were collected from under the skin on the backs of BALB/c mice, and CT26 tumors were established at the same position (*n* = 3). ImageJ was used to calculate vascular density.

### In vivo antitumor efficacy

In another batch of in vivo experiments, CT26 tumor models were established in the same manner as the 2.5 Biodistribution study. When the tumor volume reached 100 mm^3^ (day 0), the mice were randomly assigned to 6 groups: saline, SN38 sol, CPT-11, SN38 prodrug NPs (20%, 80%, and 150% NPs; *n* = 5 in each group). All of the mice in each group were injected with the appropriate material every other day for a total of 5 injections (at an equivalent SN38 concentration of 5 mg/kg). During treatment, the body weights and tumor volumes were recorded daily. A vernier caliper was used to determine the tumor volume by measuring the longest size of the tumor (length, L) and the shortest size of the tumor (width, W). Then, the tumor volume was calculated as follows: tumor volume (mm^3^) = L × W^2^ × 0.5. After treatment ended, all the mice were euthanized, and their tumors were weighed. The colorectum from each mouse was collected to determine its length. We also sampled the blood of the mice for routine blood examination. Normal BALB/c mice were used as controls (*n* = 3). After collecting blood in serum tubes, the samples were centrifuged at 4000 rpm to separate the serum, which was then used to assess liver and kidney function. Moreover, the apoptosis of the tumor cell was assessed using TUNEL antibody (Servicebio, Wuhan, China), and the proliferation of the tumor cell was assessed by Ki-67 antibody (Servicebio, Wuhan, China). ImageJ was used to calculate the quantification of the relative area (%) of apoptotic cells and proliferating cells.

### ABC effects study

BALB/c mice were injected intravenously (i.v.) with 150% NPs (at an equivalent SN38 concentration of 5 mg/kg; *n* = 3) every other day for a total of 5 injections. Normal BALB/c mice were used as controls (*n* = 3). On day 9 post-injection, blood samples were collected in serum tubes and centrifuged at 4000 rpm to separate the serum. Anti-PEG immunoglobulin M (IgM) and IgG levels were measured using the Mouse IgM ELISA kit (Elisa Lab, Wuhan Jiyinmei Biotechnology Co., Ltd., Wuhan, China) and the Mouse IgG ELISA kit (Elisa Lab, Wuhan Jiyinmei Biotechnology Co., Ltd., Wuhan, China). Complement component 3a (C3a) and C5a were measured using the Mouse C3a ELISA kit (Elisa Lab, Wuhan Jiyinmei Biotechnology Co., Ltd., Wuhan, China) and Mouse C5a ELISA kit (Elisa Lab, Wuhan Jiyinmei Biotechnology Co., Ltd., Wuhan, China).

### Statistical analysis

Analyses were performed using GraphPad Prism 10 and Microsoft Excel. All the data are presented as the means ± standard deviation (SD). The data significance between the two groups was determined with Student’s *t*-test (two-tailed). If there were more than two groups, one-way ANOVA was used. A *P*-value < 0.05 was considered statistically significant.

## Results

### Synthesis of the SN38 prodrug

To improve the self-assembly ability of SN38, we designed an SN38 prodrug by Linking 2 SN38 molecules via dodecanedioic acid. The synthesis of the SN38 prodrug was shown in Additional file 1: Fig. S1. The chemical structure of the prodrug was confirmed by MS, MS/MS, and nuclear magnetic resonance spectroscopy of hydrogen (^1^H NMR) (Additional file 1: Fig. S2a–c). MS/MS provides structural information by inducing ion fragmentation, thereby improving analytical accuracy [33]. As shown in Additional file 1: Fig. S2b, we characterized the fragment ion at *m/z* 891.26 and annotated its corresponding structure directly on the chemical diagram. The purity of the prodrug was greater than 99%, as verified by HPLC (Additional file 1: Fig. S2d).

### Preparation and characterization of the SN38 prodrug NPs

As the main component of SN38 prodrug NPs, we investigated the self-assembly ability of SN38 prodrug and compared it with that of SN38. As shown in Additional file 1: Fig. S3, SN38 readily precipitated in water and failed to form NPs, whereas the SN38 prodrug successfully formed NPs. To further investigate the self-assembly mechanism, we conducted molecular simulations to determine the binding energies and intermolecular forces between SN38 prodrug and SN38. As shown in Additional file 1: Fig. S4, the binding energy of SN38 was calculated to be −37.28 kcal/mol, whereas the SN38 prodrug exhibited a stronger binding energy of − 53.84 kcal/mol, indicating a far greater propensity for self-assembly. However, without surface engineering modification, the NPs formed solely from the SN38 prodrug (0% NPs) remained unstable, even at low concentrations (Additional file 1: Fig. S5a), and were thus excluded from further studies. The encapsulation efficiency (%) of 0% NPs was only 49.63%, indicating poor NPs formation in the absence of PEGylation. The zeta potential of 0% NPs was only − 14.60 mV.

To improve the SN38 prodrug NPs’ stability, we introduced PEGylation as a surface engineering strategy and systematically investigated the impact of the PEGylation level on SN38 prodrug NPs. SN38 prodrug NPs were prepared with various DSPE-mPEG_2k_/prodrug mass ratios (5%, 20%, 40%, 60%, 80%, 100%, 150%, and 200%), designated 0%, 5%, 20%, 40%, 60%, 80%, 100%, 150%, and 200% NPs, respectively.

**Fig. 1:**
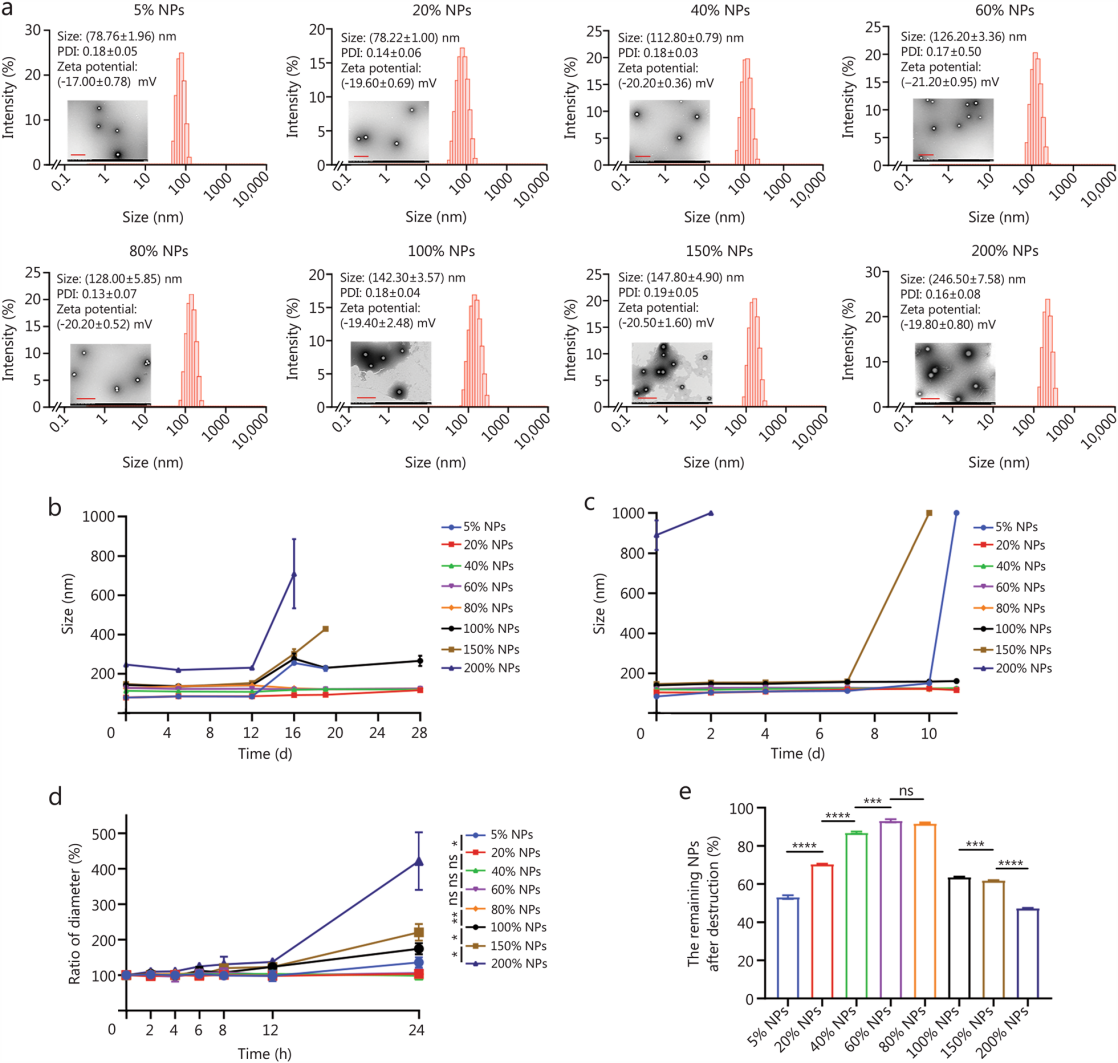
In vitro stability of the SN38 prodrug NPs. **a** Particle sizes, polydispersity index (PDI), zeta potentials, and TEM images of the SN38 prodrug NPs. Scale bar = 500 nm. Storage stability of the SN38 prodrug NPs at concentrations of 0.1 mg/ml (**b)** and 0.4 mg/ml (**c)**. **d** Stability of SN38 prodrug NPs in the presence of FBS. **e** Stability of the SN38 prodrug NPs after centrifugal destruction (*n* = 3). ^*^P < 0.05, ^**^P < 0.01, ^***^P < 0.001, ^****^P < 0.0001 by two-tailed Student’s *t*-test. ns non-significant, FBS fetal bovine serum, TEM transmission electron microscopy, SN38 7-Ethyl-10-hydroxycamptothecin, NPs nanoparticles

As shown in Additional file 1: Fig. S5, PEGylated 5–200% NPs at 0.1 mg/ml showed better Light-blue opalescence than 0% NPs, indicating the formation of better SN38 prodrug NPs. The zeta potentials of 5–200% NPs were also more negative than that of 0% NPs, suggesting enhanced surface charge due to PEGylation. Particle size progressively increased from 5 to 150% NPs, with 200% NPs reaching approximately 250 nm, indicating disrupted self-assembly at high PEGylation level (Fig. 1a). The results of the encapsulation efficiency analyses indicated that all the PEGylated SN38 prodrug NPs achieved high encapsulation efficiency while maintaining structural integrity at 0.1 mg/ml (Additional file 1: Table S1). TEM confirmed that all the SN38 prodrug NPs had spherical morphologies (Fig. 1a). We subsequently investigated the storage stability of these SN38 prodrug NPs at 0.1 mg/ml, as shown in Fig. 1b. The 5% NPs had low PEGylation, and the 100%, 150%, and 200% NPs had high PEGylation, showing instability with their increased particle size.

At a higher concentration of 0.4 mg/ml, the 200% NPs failed to form stable SN38 prodrug NPs and precipitated in deionized water. The 20–150% NPs showed better Light-blue opalescence than 5% NPs, indicating the formation of better SN38 prodrug NPs (Additional file 1: Fig. S5). Particle size also progressively increased from 5 to 150% NPs. The 5–150% NPs had high encapsulation efficiency at 0.4 mg/ml (Additional file 1: Table S2). Similarly, the 5% and 150% NPs were unstable and precipitated during storage (Additional file 1: Fig. S5), confirming the influence of the PEGylation level on the stability and self-assembly behavior of the SN38 prodrug NPs (Fig. 1c).

Stability under physiological conditions is essential for the effective delivery and cellular uptake of SN38 prodrug NPs. We further investigated the stability of the SN38 prodrug NPs in PBS (pH = 7.4) containing 30% FBS (v/v) (Fig. 1d). The SN38 prodrug NPs with PEGylation levels of 20%, 40%, 60%, and 80% showed better stability, with negligible changes in particle size after 24 h. In contrast, the 5%, 100%, 150%, and 200% NPs presented increased particle sizes, with the 200% NPs showing the most significant change. Centrifugal disruption assays [34] indicated that only 60% and 80% NPs maintained substantial aqueous dispersion of SN38 prodrug NPs post-centrifugation (Fig. 1e) and maintained hydrodynamic diameters (Additional file 1: Fig. S6), demonstrating superior stability. Other SN38 prodrug NPs with low PEGylation (5%, 20%, and 40%) or high PEGylation (100%, 150%, and 200%) displayed varying degrees of instability.

### In vitro behavior of the SN38 prodrug NPs

**Fig. 2:**
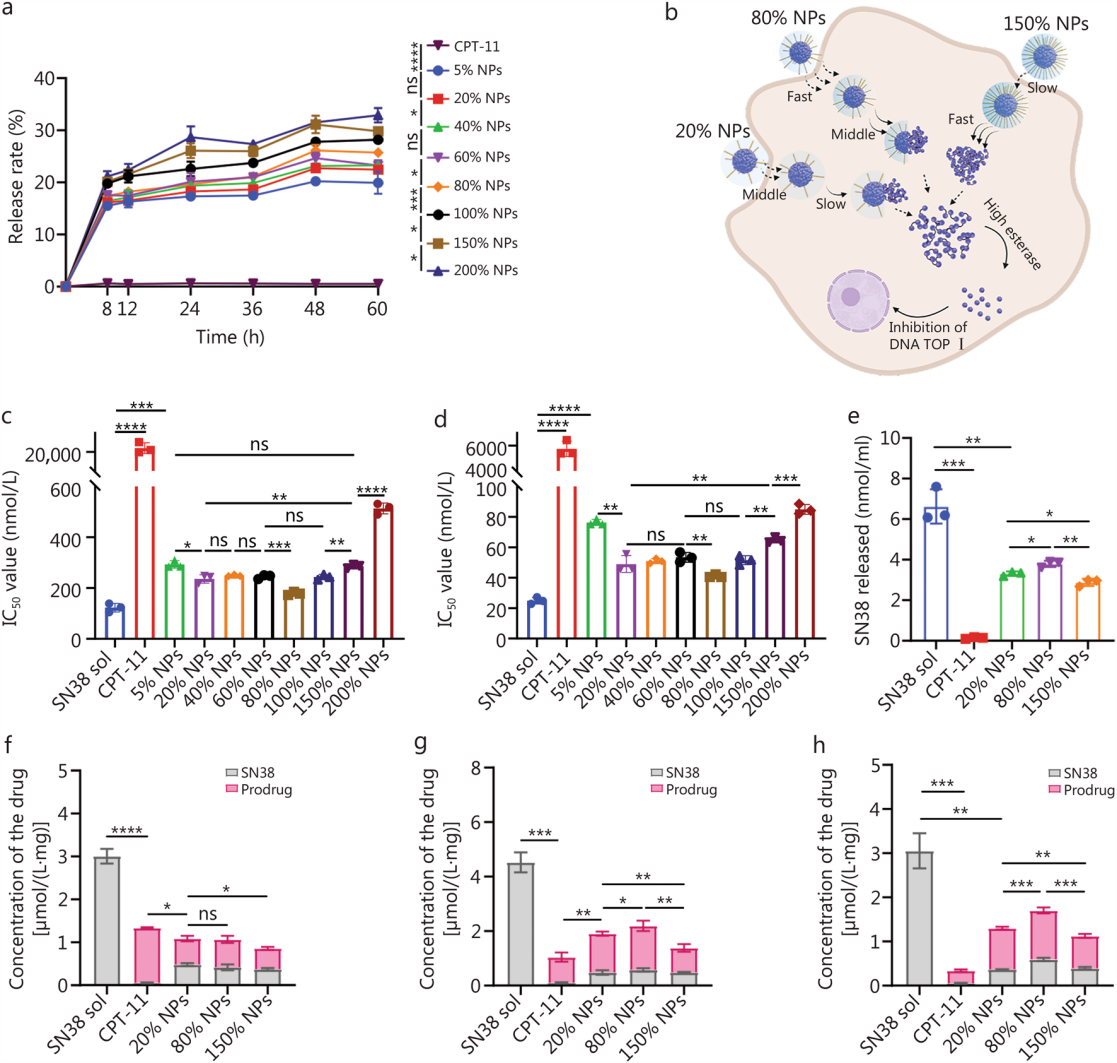
Drug release, cytotoxicity, and cellular uptake of the SN38 sol, CPT-11, and SN38 prodrug NPs. **a** Drug release from the SN38 prodrug NPs in the cell lysate. **b** Schematic of drug release and cellular uptake. The IC_50_ values of SN38 sol, CPT-11, and SN38 prodrug NPs in CT26 cells (**c**) and 4T1 cells (**d**). **e** Intracellular drug release from the SN38 prodrug NPs. The sum of SN38 and prodrug cellular uptake of the SN38 sol, CPT-11, and SN38 prodrug NPs after 2 h (**f**), 6 h (**g**), and 12 h (**h**) (*n* = 3). The *P*-value in (**f–h**) was used to compare the sum of SN38 and prodrug. ^*^P < 0.05, ^**^P < 0.01, ^***^P < 0.001, ^****^P < 0.0001 by two-tailed Student’s *t*-test. ns non-significant, SN38 7-Ethyl-10-hydroxycamptothecin, CPT-11 irinotecan, SN38 sol SN38 solution, NPs nanoparticles

We examined the release kinetics of SN38 prodrug NPs and CPT-11 using CT26 cell lysates as the release media (Fig. 2a, b). The results indicated that higher levels of PEGylation accelerated SN38 release. This acceleration is likely due to the enhanced surface hydrophilicity of the SN38 prodrug NPs upon PEGylation, which facilitates the penetration of the release medium and thereby expedites SN38 release [35]. Notably, CPT-11 showed negligible SN38 release in tumor cell lysates, reflecting its limited capacity to liberate SN38 within tumor cells.

As shown in Fig. 2c, d; Additional file 1: Fig. S7 and Table S3, we subsequently investigated the effects of various PEGylation levels on the cytotoxicity of the SN38 prodrug NPs. Among the SN38 prodrug NPs, cytotoxicity assays revealed that 80% NPs exhibited the strongest cytotoxic effect, and 200% NPs exhibited the weakest cytotoxicity. This discrepancy between cytotoxicity and drug release trend suggested that cytotoxicity may be closely related to the intracellular release rate and cellular uptake of SN38 prodrug NPs rather than just the drug release kinetics.

On the basis of the stability experiments, cellular release studies, and cytotoxicity assays, we categorized the SN38 prodrug NPs into 3 groups according to their PEGylation levels. (1) Low PEGylation (5%, 20%, and 40% NPs): these NPs exhibited insufficient stability, slower drug release rates, and moderate cytotoxicity. (2) Moderate PEGylation (60% and 80% NPs): this group demonstrated excellent stability and moderate drug release, and the 80% NPs exhibited the strongest cytotoxicity. (3) High PEGylation (100%, 150%, and 200% NPs): over PEGylation limits NPs’ stability and increases the drug release rate, but results in weaker cytotoxicity. For subsequent experiments, we selected representative SN38 prodrug NPs from each group, 20%, 80%, and 150% NPs, to further investigate their cellular uptake and in vivo behavior.

For SN38 prodrugs, cytotoxicity could only be exerted upon the release of SN38. Since the observed cytotoxicity trend differed from the drug release profile, we further quantified the intracellular release of SN38 under the same incubation conditions used for the cytotoxicity assays. The intracellular release of SN38 from these SN38 prodrug NPs aligned with their cytotoxicity profiles but did not follow the previous drug release rate trends (Fig. 2e). The SN38 prodrug NPs must enter the cell before the prodrug can be metabolized by intracellular esterase to release SN38 and exert cytotoxic effects. The intracellular release of SN38 suggested that the cellular uptake of the SN38 prodrug NPs might significantly influence intracellular drug release. PEGylation not only modulates surface properties but also directly impacts the NPs’ size and stability [36]. Therefore, PEGylation levels, colloidal stability, and particle size codetermine cellular uptake behavior of NPs [37–39]. We further investigated the cellular uptake, as shown in Figs. 1a and 2f. Even though the 80% NPs were larger than the 20% NPs, the 80% NPs exhibited the highest cellular uptake (Fig. 2f), likely due to their optimal level of PEGylation and stability (Fig. 1d, e), facilitating enhanced internalization. In contrast, despite their smaller size, the 20% NPs demonstrated weak cellular uptake (Fig. 2f–h), likely due to insufficient stability (Fig. 1d, e), even though their lower PEGylation level reduced surface hydrophilicity. High PEGylation may result in larger particle size and reduced stability, which in turn can diminish cellular uptake. Consistently, 150% NPs, which had the largest particle size (Fig. 1d, e) and high PEGylation, exhibited the lowest cellular uptake. In our system, 80% NPs achieved an optimal balance: moderate PEGylation level ensured effective protection while maintaining appropriate particle size (Fig. 1a) and enhanced stability (Fig. 1c), resulting in the highest cellular uptake (Fig. 2f). Overall, the cytotoxicity of the SN38 prodrug NPs was influenced by both the cellular uptake efficiency and drug release kinetics. However, in this system, cellular uptake appeared to be the predominant factor governing cytotoxicity.

### In vivo behavior of the SN38 prodrug NPs

**Fig. 3:**
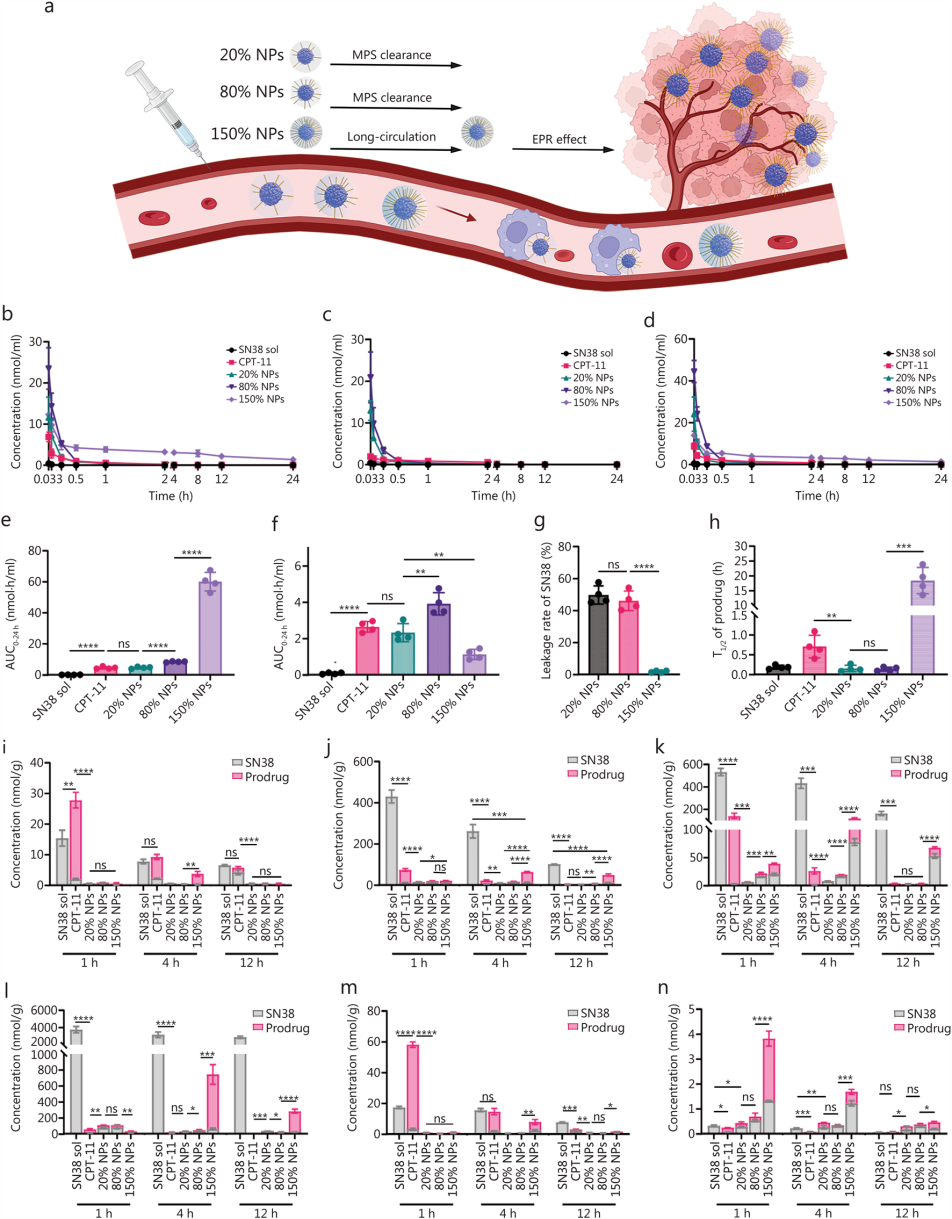
Pharmacokinetics and biodistribution behavior of the SN38 sol, CPT-11, and SN38 prodrug NPs. **a** Schematic of the pharmacokinetics and biodistribution. Molar concentration–time curves of the prodrug (**b)**, the released SN38 (**c)**, and their sum (**d)**. The AUC of the sum of SN38 and prodrug (**e)** and the released SN38 (**f)**. **g** SN38 leakage rate. **h** T_1/2_ of the prodrug (*n* = 4). Heart (**i)**, liver (**j)**, spleen (**k)**, lung (**l)**, kidneys (**m)**, and tumor (**n)** accumulation of SN38 sol, CPT-11, and the SN38 prodrug NPs (*n* = 3). The *P*-value in (**n**) was used to compare the sum of SN38 and prodrug. ^*^P < 0.05, ^**^P < 0.01, ^***^P < 0.001, ^****^P < 0.0001 by two-tailed Student’s *t*-test. ns non-significant, SN38 7-Ethyl-10-hydroxycamptothecin, CPT-11 irinotecan, SN38 sol SN38 solution, NPs nanoparticles, T_1/2_ half-life, MPS mononuclear phagocyte system, EPR enhanced permeability and retention

For small-molecule hydrophobic SN38 prodrug NPs, surface engineering via PEGylation not only influences stability, cellular uptake, release kinetics, and cytotoxicity, but also significantly impacts in vivo behavior by preventing phagocytosis by the MPS (Fig. 3a).

To precisely characterize the pharmacokinetic profiles and biodistribution, we used LC–MS-8060 for quantitative analysis. As shown in Fig. 3b–d and Additional file 1: Table S4, the blood concentrations of SN38 sol and CPT-11 declined rapidly, and the area under the curve (AUC) values of SN38 and SN38 prodrug were lower, indicating rapid clearance from the bloodstream. In contrast, the SN38 prodrug NPs exhibited significantly improved pharmacokinetic performance, with sustained blood concentrations and higher AUC values. Despite superior stability and cytotoxicity, the 80% NPs did not exhibit significant advantages compared with 150% NPs in vivo. The pharmacokinetic profiles of the 20% NPs and 80% NPs were markedly inferior to those of the 150% NPs. The sum of SN38 and prodrug AUC of the 150% NPs was 12.97 times that of CPT-11 and 7.09 times that of 80% NPs. This was Likely due to adequate PEGylation of the 150% NPs, which minimized MPS clearance and greatly enhanced systemic circulation stability (Fig. 3e). Moreover, 150% NPs effectively protected the prodrug from esterase degradation in the blood, with less than 2% SN38 release in the circulation (Fig. 3f, g). In contrast, the 20% and 80% NPs were rapidly cleared by the MPS and released nearly 50% of their SN38 payload, leading to increased toxic side effects (Fig. 3b–d; Additional file 1: Table S4). Additionally, the 150% NPs achieved a prolonged half-life of 18.43 h, whereas the 20% and 80% NPs exhibited shorter half-lives, which were even shorter than that of CPT-11 (Fig. 3h; Additional file 1: Table S4). In essence, surface engineering via PEGylation successfully addressed the instability and rapid clearance of the SN38 prodrug in the bloodstream. The 150% NPs demonstrated significantly improved pharmacokinetics, including an enhanced AUC, superior systemic stability, minimal premature drug release, and an extended half-life. These findings underscore the importance of optimizing surface engineering to improve the design of SN38 prodrug NPs for more effective cancer therapy.

Furthermore, the biodistribution of SN38 and SN38 prodrug levels in major organs was quantified following i.v. injection (Fig. 3i–n). Both SN38 sol and CPT-11 demonstrated rapid distribution to normal organs within 1 h. High concentrations of SN38 sol and CPT-11 were observed in critical organs such as the heart and kidneys, raising potential safety concerns. In contrast, compared with SN38 sol and CPT-11, tumor accumulation of the SN38 prodrug NPs was significantly improved at 1, 4, and 12 h postinjection (Fig. 3n). Among the nanoformulations, the 150% NPs presented the most favorable pharmacokinetic profile (Fig. 3e), showing superior tumor accumulation (Fig. 3n), likely due to enhanced permeability and retention (EPR) effects. We also investigated vascular distribution and density between normal tissue and CT26 tumor tissue with CD31 immunofluorescent staining (Additional file 1: Fig. S8). Quantitative analysis revealed significantly higher vascular density in tumor tissue compared to normal tissue (Additional file 1: Fig. S8), correlating with enhanced EPR-mediated NPs accumulation [40]. This enhanced accumulation was expected to improve the in vivo antitumor efficacy. At 1 h post-injection, the accumulation of the 150% NPs in the tumors was 15.32 times that of CPT-11 and 5.44 times that of the 80% NPs. To assess tumor targeting, we quantified tumor-to-organ ratios for 150% NPs (Additional file 1: Fig. S9). We also calculated the tumor-to-liver and tumor-to-spleen ratios. As the central organs of the MPS, the liver and spleen work in concert to clear NPs from systemic circulation, resulting in predominant accumulation in these organs regardless of their physicochemical properties [41]. At 1 h post-injection, 150% NPs demonstrated superior targeting with tumor-to-organ (nearly 10%), tumor-to-liver (nearly 50%), and tumor-to-spleen (nearly 100%) ratios exceeding other groups, confirming preferential tumor accumulation. However, these ratios decreased significantly at 4 h and 12 h, consistent with the stealth yet slow clearance properties characteristic of PEGylated NPs [42]. These results highlight the critical role of PEGylation in enhancing tumor accumulation, indicating its potential for generating more effective cancer therapies.

### Antitumor efficacy and safety of the SN38 prodrug NPs

**Fig. 4:**
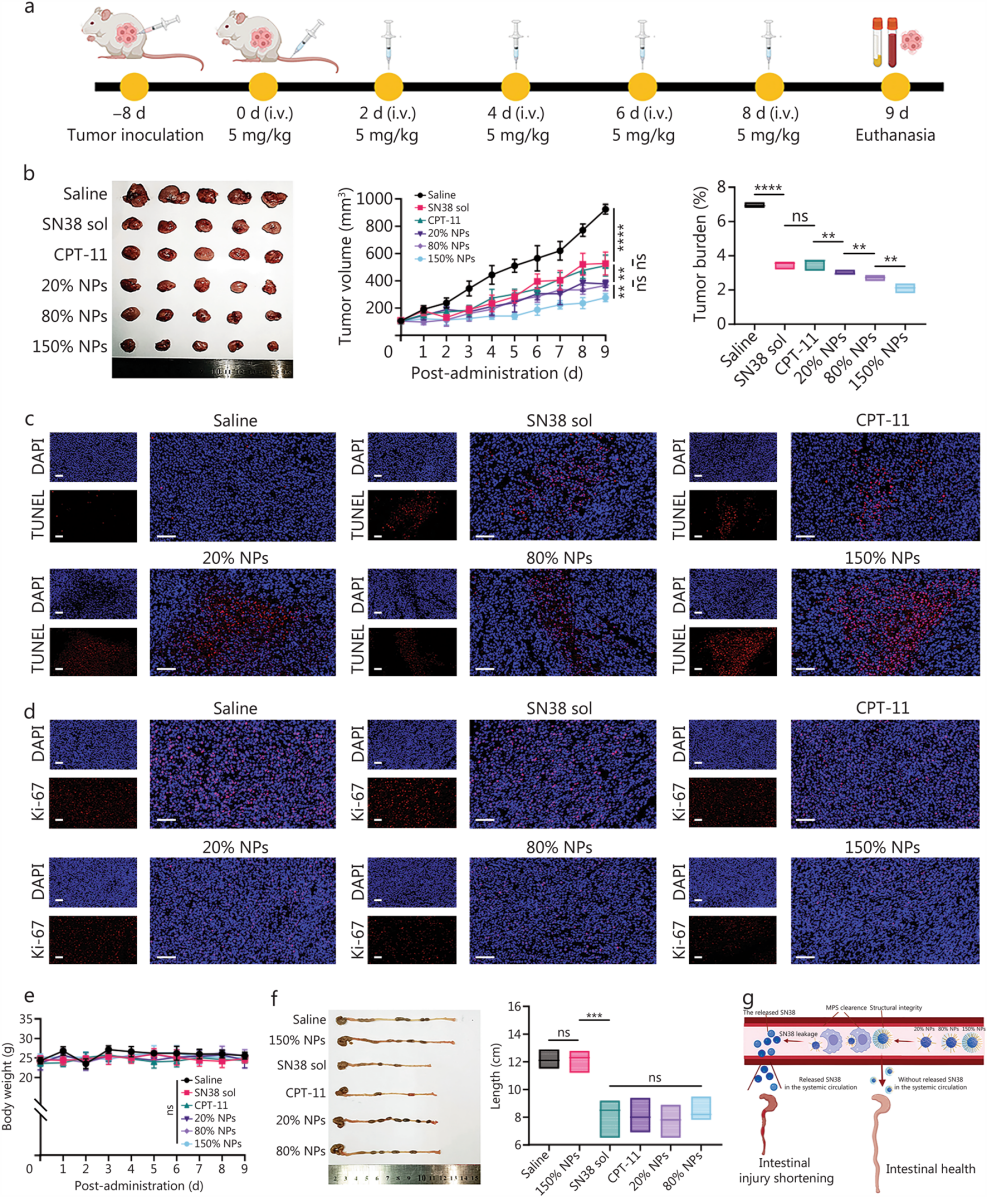
Anti-tumor efficacy and safety of the SN38 sol, CPT-11, and SN38 prodrug NPs. **a** Protocol of administering SN38 sol, CPT-11, and the SN38 prodrug NPs to BALB/c mice bearing CT26 xenografts at an SN38 dosage equivalent to 5 mg/kg. **b** Tumor photographs, volume, and burden (*n* = 5). ^**^P < 0.01, ^***^P < 0.001 and ^****^P < 0.0001 by two-tailed Student’s *t*-test. **c** TUNEL (for identifying apoptotic cells) assay images (*n* = 3). Scale bar = 50 μm. **d** Ki-67 (for indicating the proliferating cells) assay images (*n* = 3). Scale bar = 50 μm. **e** Body weight (*n* = 5). **f** Lengths and photographs of the colorectum samples (*n* = 3). ^***^P < 0.001 (one-way ANOVA followed by Dunnett’s multiple-comparisons test). **g** Schematic of SN38 leakage and intestinal injury. ns non-significant, SN38 7-Ethyl-10-hydroxycamptothecin, CPT-11 irinotecan, SN38 sol SN38 solution, NPs nanoparticles, DAPI 4,6-diamidino-2-phenylindol dihydrochloride

To validate that the PEGylation level of the SN38 prodrug NPs affects tumor progression in vivo, we investigated the antitumor efficacy of the SN38 prodrug NPs at an SN38 equivalent dose of 5 mg/kg. The dosing regimen was illustrated in Fig. 4a. Owing to the high degree of malignancy and rapid growth of CT26 cells, treatment was administered every other day to ensure therapeutic efficacy [43]. After 5 times, the tumor volume in the saline group reached nearly 1000 mm^3^, which was generally considered the ethical limit for animal studies. Consequently, the experiment was concluded at this time. The antitumor efficacy of each formulation was shown in Fig. 4b. The 150% NPs exhibited the most potent antitumor efficacy, which could be attributed to their effective evasion of clearance by the MPS. This allowed the 150% NPs to remain at high concentrations in blood with a prolonged half-life, ultimately resulting in greater tumor accumulation. TUNEL assays were performed to assess tumor cell apoptosis, and Ki-67 assays were used to evaluate tumor cell proliferation. The 150% NPs group exhibited the highest level of tumor apoptosis (Fig. 4c; Additional file 1: Fig. S10) and the lowest level of proliferation (Fig. 4d; Additional file 1: Fig. S10), further demonstrating the superior in vivo antitumor efficacy of the 150% NPs.

About safety, no significant weight loss was detected in any treatment group at the dosage administered (Fig. 4e). Considering the specific intestinal toxicity associated with SN38 [44], we utilized colorectal length to evaluate the safety profile (Fig. 4f). Compared with that in the saline group, only the colorectal length in the 150% NP group remained unaltered. Significant shortening was observed in the SN38 sol, CPT-11, 20% NPS, and 80% NPS groups, indicating severe intestinal toxicity. These findings suggested that the 150% NPs, equipped with sufficient PEGylation, remained stable during systemic circulation, thereby preventing the premature release of SN38 and mitigating SN38 toxicity. In contrast, 20% NPs and 80% NPs demonstrated considerable SN38 leakage in the bloodstream, leading to considerable intestinal side effects (Fig. 4g). Furthermore, routine hematological assessments, as well as liver and kidney function tests, revealed no significant toxicity across all groups, underscoring the safety of the surface-engineering SN38 prodrug NPs (Additional file 1: Fig. S11).

We also focused on the risk of ABC effects, a common concern for PEGylated NPs [45]. As shown in Additional file 1: Fig. S12, no significant differences in anti-PEG IgM or IgG levels were observed between the 150% NPs group and the normal control group. Additionally, levels of anaphylatoxins C3a and C5a remained comparable, indicating that 150% NPs did not trigger complement activation. These results suggested that 150% NPs have a low potential to induce immune-related ABC effects and exhibit good biocompatibility in vivo. Therefore, despite suboptimal in vitro stability and cellular behavior, the 150% NPs, benefiting from sufficient PEGylation, demonstrated superior in vivo pharmacokinetics and tumor accumulation, culminating in the best anti-tumor efficacy without ABC induction.

## Discussion

**Fig. 5:**
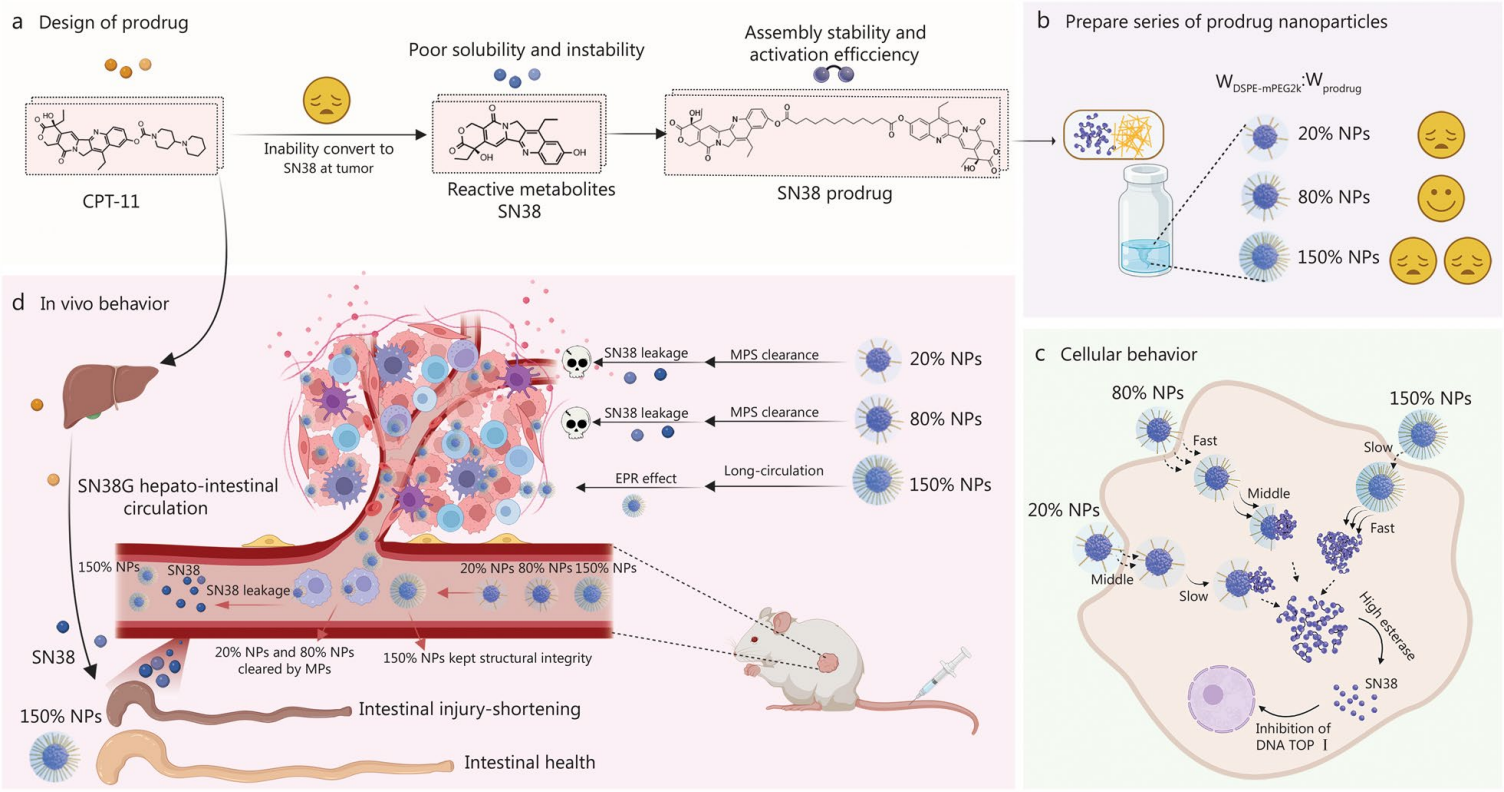
Surface engineering has dual effects on the in vitro and in vivo behavior of the SN38 prodrug NPs. **a** Design of prodrug. Due to the inability of CPT-11 to release SN38 at the tumor site and the poor solubility and stability of SN38, we have designed and developed an SN38 prodrug that can self-assemble into stable NPs and be activated at the tumor site. **b** Prepare a series of prodrug nanoparticles (NPs). 80% NPs demonstrated excellent in vitro stability. **c** Cellular behavior. 80% NPs demonstrated excellent cytotoxicity and cellular uptake. **d** In vivo behavior. 20% NPs and 80% NPs were rapidly cleared by the rapid mononuclear phagocyte system (MPS) in vivo. In contrast, the structural stability of the 150% NPs was maintained in circulation, resulting in maximum SN38 tumor accumulation and enhanced antitumor efficacy. SN38 7-Ethyl-10-hydroxycamptothecin, CPT-11 irinotecan, SN38G SN38-glucuronide, EPR enhanced permeability and retention, TOP I topoisomerase I

PEGylation forms a hydrophilic layer on NPs, improving stability by preventing aggregation and precipitation of hydrophobic small-molecule prodrugs in vitro, while also reducing protein adsorption and immune recognition in vivo. This prolongs systemic circulation and enhances tumor targeting via the EPR effect. At low PEGylation (“mushroom” regime), serum proteins can penetrate the PEG layer, triggering immune clearance, whereas a dense PEGylation “brush” effectively blocks such interactions [46]. In this study, we systematically evaluated the effects of the PEGylation level on the stability, drug release behavior, cytotoxicity, cellular uptake, pharmacokinetic profile, biodistribution, antitumor efficacy, and intestinal toxicity of SN38 prodrug NPs (Fig. 5).

Our data reflected the so-called “PEG dilemma”, the need to balance stealth properties with cellular uptake. The low PEGylation SN38 prodrug NPs (20% NPs) failed to achieve sufficient PEGylation, resulting in poor in vitro stability and rapid MPS clearance. The 80% NPs demonstrated optimal in vitro characteristics, including stability, cellular uptake, and cytotoxicity. However, 80% NPs proved insufficient in vivo, as the PEGylation shell could not fully prevent MPS clearance. In contrast, the high PEGylation 150% NPs exhibited markedly improved pharmacokinetics and tumor accumulation. The dense PEGylation provided effective immune shielding, reduced MPS clearance, and increased tumor-to-normal tissue ratios, ultimately leading to superior antitumor efficacy. Conversely, while 150% NPs showed slightly reduced cellular uptake due to high PEGylation, their prolonged circulation allowed more effective tumor accumulation, compensating for lower per-cell internalization. This suggested that for chemotherapy, poorly soluble drugs like SN38, ensuring prolonged systemic circulation and tumor accumulation is more important than maximizing cellular uptake.

We also calculated the tumor-to-organ ratios to investigate the tumor targeting of 150% NPs. Although the tumor-to-organ ratio of 150% NPs at 1 h was higher than that of other groups, the tumor-to-organ ratio was only 10%. This suggested that while 150% NPs exhibit some level of preferential tumor accumulation early on, this difference may not be substantial enough to provide a clear advantage in terms of targeting efficiency in the short term. Importantly, antitumor efficacy in the CT26 model was more closely correlated with the absolute drug accumulation in tumors [20, 47]. This finding highlights the importance of overall drug concentration within the tumor, rather than relying solely on relative accumulation when evaluating chemotherapy. In the CT26 tumor model, passive EPR targeting alone can result in substantial tumor accumulation, and surface engineering is crucial for further enhancing drug delivery, extending circulation time, and improving the efficiency of EPR targeting. In contrast, CPT-11, 20% NPs, and 80% NPs exhibited comparatively higher tumor-to-organ ratios at 4 and 12 h. This observation Likely resulted from rapid clearance of formulations with insufficient surface engineering, leading to extremely low drug concentrations in both tumors and organs beyond 1 h. Such low concentrations may amplify analytical errors during ratio calculation, limiting the reliability in demonstrating targeting enhancement [48].

ABC, caused by anti-PEG immune responses, often limits the efficacy of PEGylated systems [45]. In our research, 150% NPs did not trigger ABC effects upon repeated dosing. This absence of immunogenicity may stem from the cytotoxicity of the SN38 prodrug, which is akin to doxorubicin, that could suppress the primary producers of anti-PEG antibodies from B cells and reduce PEG immunogenicity and ABC risk [49]. The 150% NPs had a lower DSPE-mPEG_2k_/drug mass ratio (1.5:1) than Doxil® (1.6:1). However, Doxil® exhibits no ABC effects clinically due to doxorubicin’s B-cell toxicity [50]. The ABC effect is predominantly triggered by empty PEGylated liposomes [51, 52]. In contrast, our NPs formed via prodrug self-assembly, with PEGylation solely enhancing circulation time. Upon drug release, NPs disassembly prevented the prolonged circulation of PEG. The absence of ABC in this study suggested that this high PEGylation strategy may be suitable for repeated administration of cytotoxic hydrophobic drugs without inducing immunogenicity, offering a promising route for safer and more effective chemotherapy delivery.

Our study highlights that surface engineering is as critical as prodrug chemistry in the design of nanomedicines. Modulating PEGylation levels significantly influenced NPs’ stability, cellular uptake, pharmacokinetics, tumor accumulation, and antitumor efficacy. Low PEGylation led to premature clearance and reduced delivery efficiency, while high PEGylation enabled prolonged circulation and enhanced antitumor outcomes. Despite modestly reduced uptake, high PEGylation ensured effective tumor targeting and minimized systemic toxicity. These findings underscore the importance of optimizing surface properties to achieve a functional balance between circulation time and drug delivery efficiency.

## Conclusions

We developed a series of different PEGylation levels of SN38 prodrug NPs to explore the impact of surface engineering on in vitro and in vivo behavior. Low PEGylation (20% NPs) led to poor stability and rapid clearance, whereas moderate PEGylation (80%) improved stability and in vitro performance but failed to evade MPS clearance, resulting in accelerated clearance and reduced efficacy in vivo. High PEGylation (150% NPs) offered superior pharmacokinetics, tumor accumulation, and antitumor efficacy without inducing ABC effects. These results highlight the importance of optimizing surface properties to balance circulation stability and tumor delivery in nanomedicine design.

## Supplementary Information

**Additional file 1.** Methods.** Fig. S1** Synthetic route of SN38 dimeric prodrug. **Fig. S2** Structure confirmation of SN38 prodrug. **Fig. S3** Self-assembly of SN38 and SN38 prodrug in water at 0.1 mg/ml. **Fig. S4** Intermolecular interactions of SN38 and SN38 prodrug during the self-assembly process. **Fig. S5 **Stability of SN38 prodrug NPs at 0.1 mg/ml and 0.4 mg/ml. **Fig. S6** Stability of the SN38 prodrug NPs after centrifugal destruction. **Fig. S7** Cell viability of CT26 cells and 4T1 cells after treatment with various concentrations of SN38 sol, CPT-11, and SN38 prodrug NPs. **Fig. S8** Expression of CD31 in normal tissue under the skin on the backs of BALB/c mice and CT26 tumors established at the same position. **Fig. S9** Tumor-to-organ ratios, tumor-to-liver ratios, and tumor-to-spleen ratios at 1, 4, and 12 h. **Fig. S10** Fluorescence quantitative results of TUNEL assay (for identifying apoptotic cells) and Ki67 (for indicating the proliferating cells) assay. **Fig. S11** Blood routine examination and hepatorenal function parameters. **Fig. S12** Anti-PEG and anaphylatoxins responses for 150% NPs. **Table S1** Characterization of SN38 prodrug NPs (0.1 mg/ml). **Table S2** Characterization of SN38 prodrug NPs (0.4 mg/ml). **Table S3** IC50 of SN38 sol, CPT-11, and SN38 prodrug NPs. **Table S4** Pharmacokinetic profiles of SN38 sol, CPT-11, and SN38 prodrug NPs.

## Data Availability

All data supporting the findings of this study are available within the article and the Additional file. he source data underlying Figs. 1a–e, 2a, c–h, 3b–n, 4b, e–f, and Additional file 1: Figs. S6–S12, Tables S1–S2 have been deposited in the Figshare database (10.6084/m9.figshare.30074437.v1).

## Abbreviations

^1^H NMR: Nuclear magnetic resonance spectroscopy of hydrogen
ABC: Accelerated blood clearance
ADCs: Antibody-drug conjugates
AUC: Area under the curve
C3a: Complement component 3a
C5a: Complement component 5a
CD31: Platelet endothelial cell adhesion molecule-1
DAPI: 4,6-Diamidino-2-phenylindol dihydrochloride
DMAP: 4-Dimethylaminopyridine
DMF: N, N-dimethylformamide
DMSO: Dimethyl sulfoxide
DSPE-mPEG_2k_: N-(carbonyl-methoxypolyethyleneglycol-2000)-1,2-distearoyl-sn-glycerol-3-phosphoethanolamine
EDCI: 1-(3-Dimethylaminopropyl)-3-ethylcarbodiimide hydrochloride
EPR: Enhanced permeability and retention
FBS: Fetal bovine serum
FDA: Food and drug administration
HCPT: 10-Hydroxycamptothecin
HPLC: High-performance liquid chromatography
IgG: Immunoglobulin G
IgM: Immunoglobulin M
i.v.: Intravenous
LC-MS: Liquid chromatography-mass spectrometry
MPS: Mononuclear phagocyte system
MS: Mass spectrometry
MS/MS: Tandem mass spectrometry
MTT: 3-(4,5-Dimethylthiazol-2-yl)-2,5-diphenyltetrazolium bromide
NPs: Nanoparticles
PBS: Phosphate buffer saline
PDI: Polydispersity index
PEG: Polyethylene glycol
SD: Standard deviation
SN38: 7-Ethyl-10-hydroxycamptothecin
SN38 sol: SN38 solution
SN38G: SN38-glucuronide
TEM: Transmission electron microscopy
TOP I: Topoisomerase I
UGT1A1: Uridine diphosphate-glucuronosyltransferase 1A1
