# Supplementary material for: Dual character of surface engineering on SN38 prodrug nano-assemblies: divergent effects on in vitro and in vivo behavior
Source: Mil Med Res. 2025 Sep 22;12:60. doi: 10.1186/s40779-025-00648-6 (PMC12452009; doi:10.1186/s40779-025-00648-6)
Supplement: Supplementary file 1 — Additional file 1. Methods. Fig. S1 Synthetic route of SN38 dimeric prodrug. Fig. S2 Structure confirmation of SN38 prodrug. Fig. S3 Self-assembly of SN38 and SN38 prodrug in water at 0.1 mg/ml. Fig. S4 Intermolecular interactions of SN38 and SN38 prodrug during the self-assembly process. Fig. S5 Stability of SN38 prodrug NPs at 0.1 mg/ml and 0.4 mg/ml. Fig. S6 Stability of the SN38 prodrug NPs after centrifugal destruction. Fig. S7 Cell viability of CT26 cells and 4T1 cells after treatment with various concentrations of SN38 sol, CPT-11, and SN38 prodrug NPs. Fig. S8 Expression of CD31 in normal tissue under the skin on the backs of BALB/c mice and CT26 tumors established at the same position. Fig. S9 Tumor-to-organ ratios, tumor-to-liver ratios, and tumor-to-spleen ratios at 1, 4, and 12 h. Fig. S10 Fluorescence quantitative results of TUNEL assay (for identifying apoptotic cells) and Ki67 (for indicating the proliferating cells) assay. Fig. S11 Blood routine examination and hepatorenal function parameters. Fig. S12 Anti-PEG and anaphylatoxins responses for 150% NPs. Table S1 Characterization of SN38 prodrug NPs (0.1 mg/ml). Table S2 Characterization of SN38 prodrug NPs (0.4 mg/ml). Table S3 IC50 of SN38 sol, CPT-11, and SN38 prodrug NPs. Table S4 Pharmacokinetic profiles of SN38 sol, CPT-11, and SN38 prodrug NPs. [file 40779_2025_648_MOESM1_ESM.pdf]

Methods

Materials

N-(carbonyl-methoxypolyethyleneglycol-2000)-1,2-distearoyl-sn-glycerol-3-phosphoethanolamine ($DSPE-mPEG_{2k}$) was obtained from AVT (Shanghai) Pharmaceutical Tech Co., Ltd. (Shanghai, China). 7-Ethyl-10-hydroxycamptothecin (SN38), irinotecan (CPT-11), 10-hydroxycamptothecin (HCPT), 3-(4,5-dimethylthiazol-2-yl)-2,5-diphenyltetrazolium bromide (MTT), and Roswell Park Memorial Institute (RPMI) 1640, Dulbecco's modified Eagle medium (DMEM) media, penicillin, and streptomycin were obtained from Meilun Biotechnology Co., Ltd. (Dalian, China). Dodecanedioic acid 1-(3-dimethylaminopropyl)-3-ethylcarbodiimide hydrochloride (EDCI, 99%) and 4-dimethylaminopyridine (DMAP, 99%) were provided by Macklin Biochemical Technology Co., Ltd. (Shanghai, China). The 96-well, 24-well, 12-well, and 6-well plates were purchased from NEST Biotechnology Co., Ltd. (Wuxi, China). The BCA assay kit was obtained from Beijing Solarbio Science & Technology Co., Ltd. (Beijing, China).

Synthesis of the SN38 prodrug and preparation of the SN38 prodrug nanoparticles (NPs) with different ratios of $DSPE-mPEG_{2k}$

The 0.4 mmol SN38 and 0.2 mmol aliphatic dibasic acid were dissolved in N, N-dimethylformamide (DMF). Then, 0.04 mmol DMAP and 0.8 mmol EDCI were added as catalysts for ester bond formation. After 12 h, 0.04 mmol DMAP and 0.4 mmol EDCI were also added, and the reaction continued for 24 h. The SN38 prodrug was purified by preparative liquid chromatography and characterized via nuclear magnetic resonance spectroscopy of hydrogen (400 MHz ^{1}H NMR, AV-400, Bruker Corporation, Germany), low-resolution mass spectrometry (MS, ACQUITYQDa, Waters Co., Ltd., USA), ultra-high performance liquid chromatography/tandem mass spectrometry (UPLC-MS/MS) (Waters Co., Ltd., USA) and high-performance liquid chromatography (HPLC) (Hitachi, Japan).

SN38 prodrug NPs (0 – 200% NPs) were prepared using a one-step nanoprecipitation method. Briefly, for 0.1 mg/ml 0% NPs, SN38 prodrug was dissolved at a concentration of 1 mg/ml in 200 μl of tetrahydrofuran (THF) containing 25% DMF. The organic solution was added dropwise to 2 ml of deionized water under vigorous stirring to facilitate NP formation. The organic solvent was removed by rotary evaporation. For 5 – 200% NPs, the same procedure was followed, with $DSPE-mPEG_{2k}$ added to the organic phase at specific $DSPE-mPEG_{2k}$-to-prodrug mass ratios (5 – 200%). In addition,

SN38 was dissolved at 1 mg/ml in THF (25% DMF) and added dropwise to 2 ml of deionized water under vigorous stirring to examine SN38 self-assembly behavior. The encapsulation efficiency was determined by gel filtration chromatography. The drug loading of the SN38 prodrug NPs was calculated as: drug loading (%) = $W_{SN38}/(W_{prodrug} + W_{DSPE\text{-}mPEG2k})$ × encapsulation efficiency × 100%.

Liquid chromatography-mass spectrometry (LC-MS) method

We used LC-MS-8060 (Shimadzu Corporation, Japan) to quantify SN38 sol, CPT-11, and SN38 prodrug NPs in various biological samples, including cells, plasma, and tissues. We chose HCPT as an internal standard. The chromatographic column used was a Desc. Kinetex 2.6 μm XB-C18 100A column (part No. 00B-4496-AN) with a size of 50 mm × 2.1 mm. Additionally, the flow rate was 0.4 ml/min, and the mobile phase was composed of acetonitrile and deionized water (containing 0.2% methanoic acid). The MS target ions and parameters were as follows: HCPT *m/z* 364.5, 321.25, cone voltage: –24 mV; SN38 *m/z* 392.5, 349.25, cone voltage: –26 mV; CPT-11 *m/z* 587.4, 124.2, cone voltage: –37 mV; and SN38 prodrug *m/z* 979.4, 349.10, cone voltage: –55 mV.

Drug release study

CT26 cells were used for the drug release study. The cells were broken in purified water and diluted to a concentration of 6.6×10^5 cells/ml. Then, 5% (V/V) dimethyl sulfoxide (DMSO) was added to the above media. Subsequently, 5 – 200% NPs and CPT-11 (at an equivalent SN38 concentration of 68 μmol/L, $C_{SN38\ prodrug\ at\ an\ equivalent\ SN38\ concentration}$ = Molecular weight of SN38 in the SN38 prodrug/Molecular weight of SN38 prodrug × $C_{SN38\ prodrug}$) were added to the corresponding release media. All of the samples were maintained at 37 °C with shaking. At 8, 12, 24, 48, and 60 h, 50 μl of the medium was taken, and 150 μl of methanol was added to precipitate the protein. The cell sample with methanol was centrifuged (13,000 rpm, 10 min), and the supernatant was collected. HPLC was used for quantitative analysis of the released SN38. The drug release rate was calculated as follows: drug release rate (%) = peak area of released SN38 in sample medium/peak area of the theoretical amount of SN38 × C_{SN38} × 100%. All of the above samples were maintained at 37 °C with shaking at 100 r/min.

Cytotoxicity study

CT26 and 4T1 cells were plated in 96-well plates at a density of 1000 cells per well and incubated for 24 h. Then, the media was replaced with new media containing different concentrations of SN38

solution (SN38 sol), CPT-11, or SN38 prodrug NPs (at an equivalent SN38 concentration). For CT26 cells, the concentrations were treated as follows: SN38 sol and SN38 prodrug NPs were 5, 10, 20, 50, 100, 200, 500, 1000, and 2000 nmol/L; CPT-11 was 500, 1000, 2000, 5000, 10,000, 20,000, 40,000, and 80,000 nmol/L. For 4T1 cells, the concentrations were treated as follows: SN38 sol and SN38 prodrug NPs were 1, 5, 10, 20, 50, 100, 200, 500, and 1000 nmol/L; CPT-11 was 200, 500, 1000, 2000, 5000, 10,000, 20,000, 40,000, and 80,000 nmol/L. After 48 h of culture, 20 μl of 5 mg/ml MTT solution was added to each well, and cultivation was continued for 4 h. The crystals at the bottoms of the wells of the 96-well plates were dissolved in 200 μl of DMSO. Finally, the absorbance of each well was measured with a microplate reader (Thermo, USA).

Cellular uptake study

CT26 cells were cultured in 6-well plates (2×10^5 cells per well) for 48 h. The new medium containing SN38 sol, CPT-11, or SN38 prodrug NPs (at an equivalent SN38 concentration of 15 μmol/L) was used to culture the cells, and the culture continued for 2, 6, or 12 h. Next, we removed the drug-containing media, washed the cells with cold PBS, and lysed the cells. The protein concentration was quantified with the BCA kit (Beijing Solarbio Science & Technology Co., Ltd., China). LC-MS-8060 was used to investigate the concentration of SN38 sol, CPT-11, and SN38 prodrug NPs in cellular uptake samples.

Intracellular release study

CT26 cells were seeded into 6-well plates at a density of 2×10^5 cells per well and incubated for 48 h. Then, SN38 sol, CPT-11, or SN38 prodrug NPs (at an equivalent SN38 concentration of 15 μmol/L) were added for 48 h of incubation, after which time the release of SN38 in each group was evaluated. After sonication and centrifugation, the concentrations of free SN38 in the supernatants were determined by HPLC.

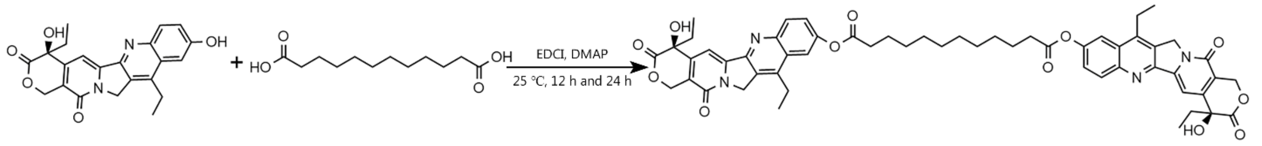

Fig. S1 Synthetic route of SN38 dimeric prodrug. SN38 7-Ethyl-10-hydroxycamptothecin, EDCI 1-(3-dimethylaminopropyl)-3-ethylcarbodiimide hydrochloride, DMAP 4-dimethylaminopyridine

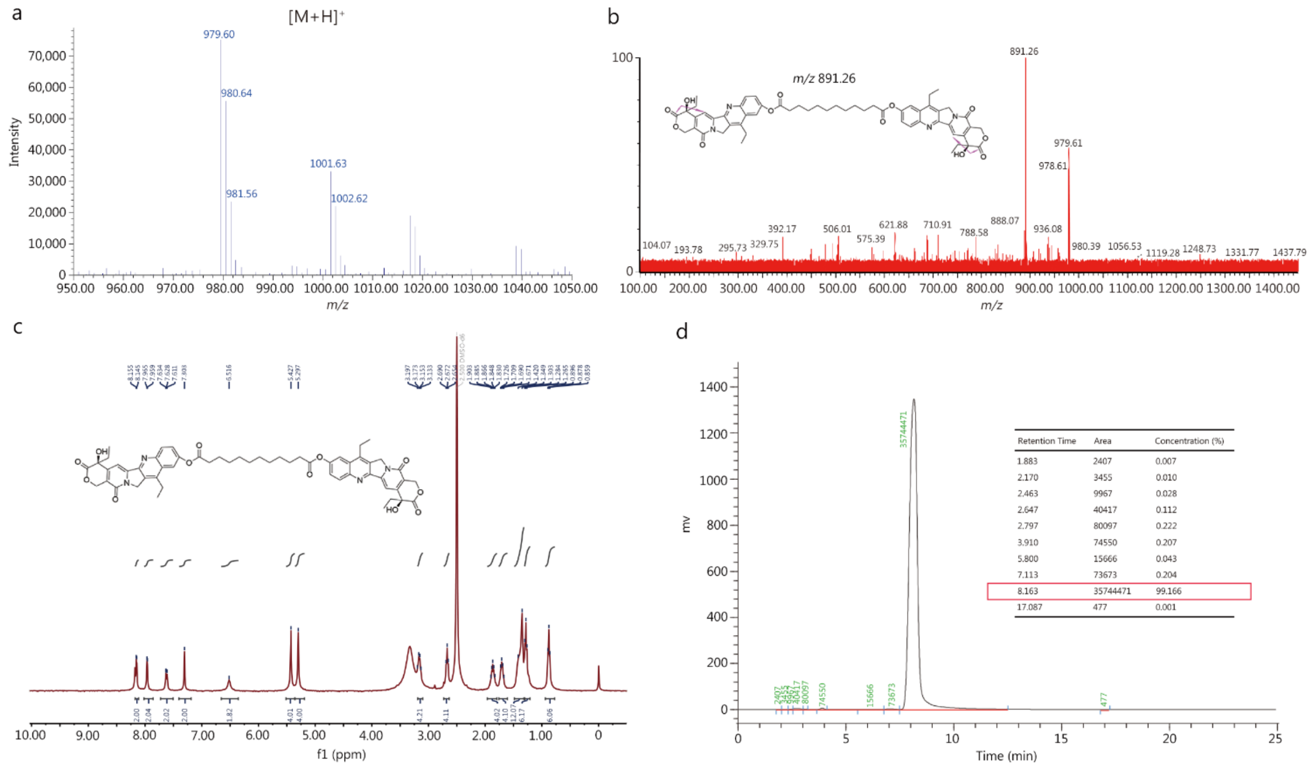

Fig. S2 Structure confirmation of SN38 prodrug. **a** MS. **b** MS/MS. The fragment ion structure in the MS/MS spectrum was highlighted with pink arrows. **c** ^{1}H NMR. **d** HPLC. ^{1}H NMR (400 MHz, DMSO) δ 8.15 (d, J = 3.8 Hz, 2H), 8.02 – 7.86 (m, 2H), 7.73 – 7.51 (m, 2H), 7.30 (s, 2H), 6.52 (s, 2H), 5.43 (s, 4H), 5.30 (s, 4H), 3.16 (d, J = 8.0 Hz, 4H), 2.67 (t, J = 7.3 Hz, 4H), 1.95 – 1.81 (m, 4H), 1.75 – 1.69 (m, 4H), 1.49 – 1.32 (m, 12H), 1.28 (t, J = 7.5 Hz, 6H), 0.88 (t, J = 7.3 Hz, 6H). MS mass spectrometry, MS/MS tandem mass spectrometry, ^{1}H NMR nuclear magnetic resonance spectroscopy of hydrogen, HPLC high-performance liquid chromatography, SN38 7-Ethyl-10-hydroxycamptothecin, DMSO dimethyl sulfoxide, *m/z* Mass-to-charge ratio

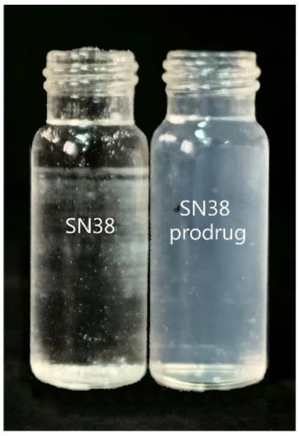

Fig. S3 Self-assembly of SN38 and SN38 prodrug in water at 0.1 mg/ml. SN38 7-Ethyl-10-hydroxycamptothecin

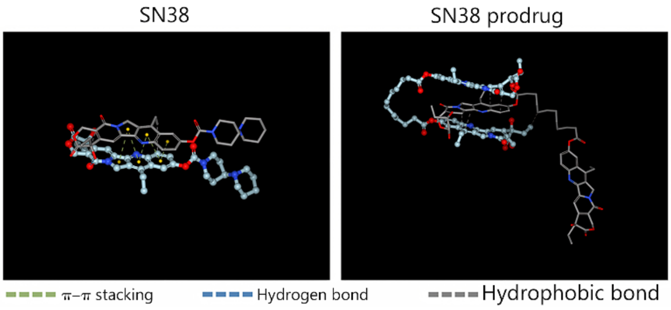

Fig. S4 Intermolecular interactions of SN38 and SN38 prodrug during the self-assembly process. SN38 7-Ethyl-10-hydroxycamptothecin

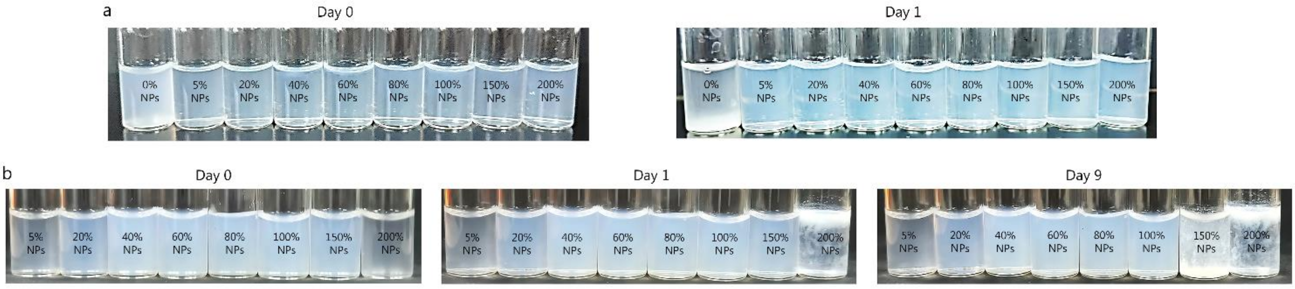

Fig. S5 Stability of SN38 prodrug NPs at 0.1 mg/ml (**a**) and 0.4 mg/ml (**b**). NPs nanoparticles, SN38 7-Ethyl-10-hydroxycamptothecin

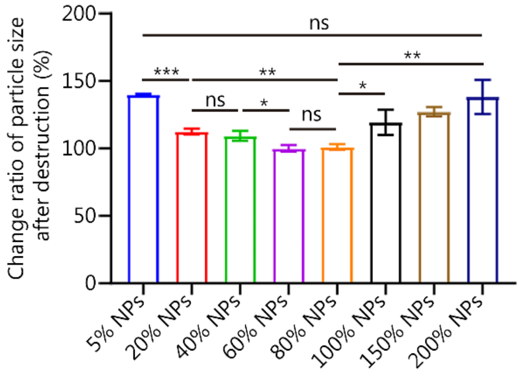

Fig. S6 Stability of the SN38 prodrug NPs after centrifugal destruction (n = 3). $^{*}P < 0.05$, $^{**}P < 0.01$, $^{***}P < 0.001$ by two-tailed Student's t-test. ns non-significant, NPs nanoparticles, SN38 7-Ethyl-10-hydroxycamptothecin

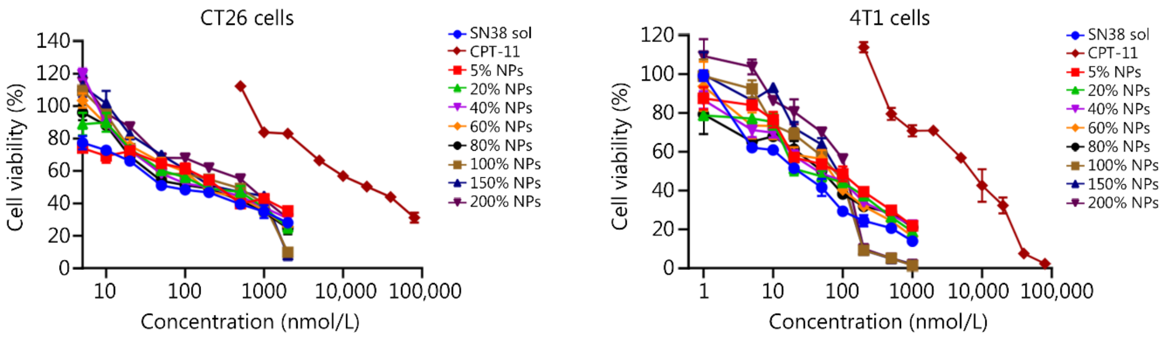

Fig. S7 Cell viability of CT26 and 4T1 cells after treatment with various concentrations of SN38 sol, CPT-11, and SN38 prodrug NPs (n = 3). SN38 7-Ethyl-10-hydroxycamptothecin, CPT-11 irinotecan, SN38 sol SN38 solution, NPs nanoparticles

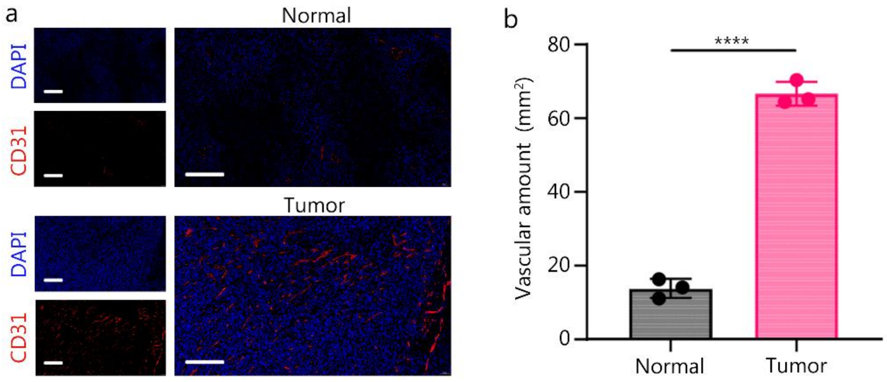

Fig. S8 Expression of CD31 in normal tissue under the skin on the backs of BALB/c mice and CT26 tumors established at the same position. **a** Immunofluorescence staining images of normal and tumor tissues. **b** Quantitative analysis of vascular density in normal tissues vs. tumor tissues ($n = 3$). Scale bar = 50 μm. $^{****}P < 0.0001$ by two-tailed Student's t-test. DAPI 4,6-diamidino-2-phenylindol dihydrochloride, CD31 platelet endothelial cell adhesion molecule-1

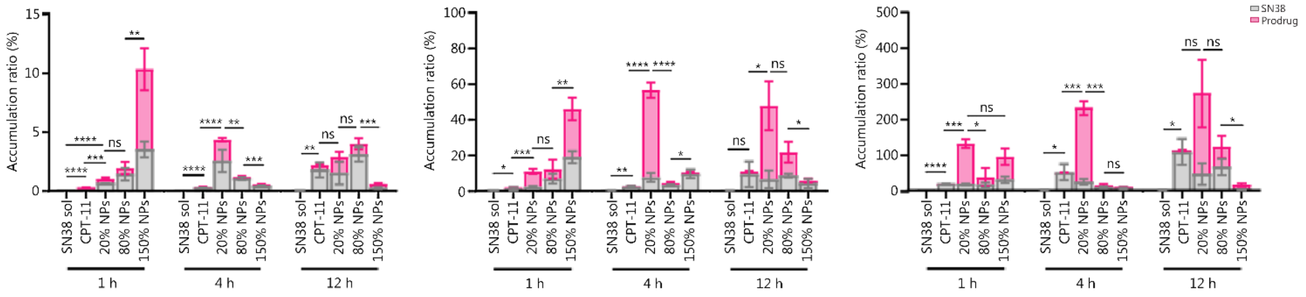

Fig. S9 Tumor-to-organ ratios, tumor-to-liver ratios, and tumor-to-spleen ratios at 1, 4, and 12 h (n = 3). The P-value was used to compare the sum of SN38 and the prodrug. $^{*}P < 0.05$, $^{**}P < 0.01$, $^{***}P < 0.001$, $^{****}P < 0.0001$ by two-tailed Student's t-test. ns non-significant, SN38 7-Ethyl-10-hydroxycamptothecin, CPT-11 irinotecan, SN38 sol SN38 solution, NPs nanoparticles

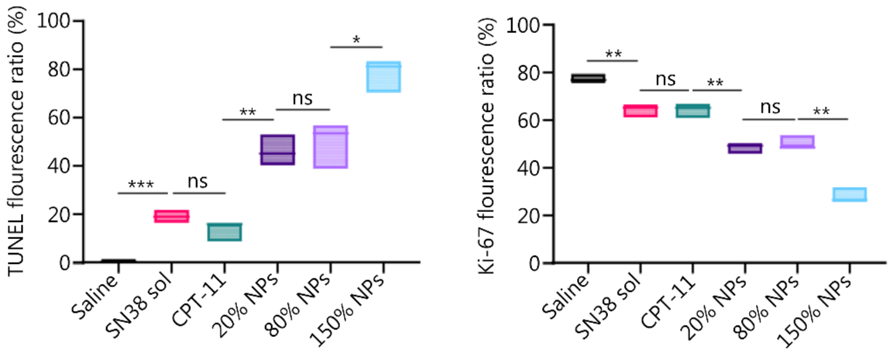

Fig. S10 Fluorescence quantitative results of TUNEL assay (for identifying apoptotic cells) and Ki-67 (for indicating the proliferating cells) assay (n = 3). $^{*}P < 0.05$, $^{**}P < 0.01$, $^{***}P < 0.001$ by two-tailed Student's *t*-test. ns non-significant, SN38 7-Ethyl-10-hydroxycamptothecin, CPT-11 irinotecan, SN38 sol SN38 solution, NPs nanoparticles

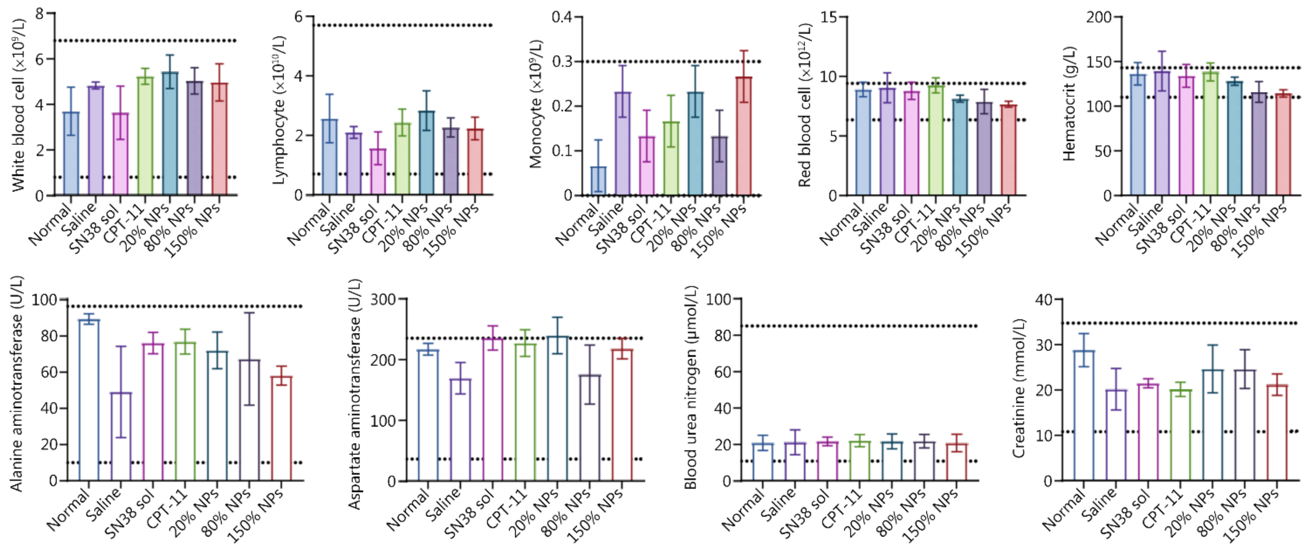

Fig. S11 Blood routine examination and hepatorenal function parameters (*n* = 3). SN38 7-Ethyl-10-hydroxycamptothecin, CPT-11 irinotecan, SN38 sol SN38 solution, NPs nanoparticles

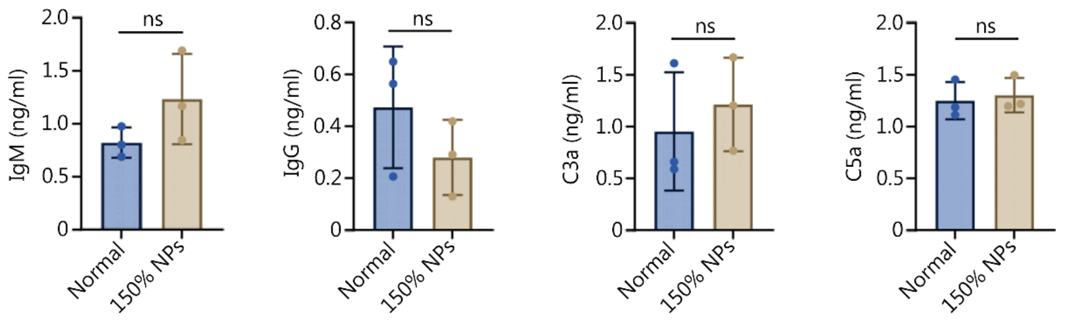

Fig. S12 Anti-PEG and anaphylatoxins responses for 150% NPs (n = 3). P-values were calculated by a two-tailed Student's t-test. ns non-significant, NPs nanoparticles, PEG polyethylene glycol, IgM immunoglobulin M, IgG immunoglobulin G, C3a complement component 3a, C5a complement component 5a

Table S1 Characterization of SN38 prodrug NPs (0.1 mg/ml) (%, *n* = 3, mean ± SD)

**Formulations**	**Encapsulation efficiency**	**Drug loading (w/w)**
5% NPs	96.48 ± 0.47	73.51 ± 0.36
20% NPs	95.17 ± 0.13	63.45 ± 0.09
40% NPs	96.69 ± 0.38	55.25 ± 0.22
60% NPs	95.86 ± 0.12	47.93 ± 0.06
80% NPs	97.92 ± 0.07	43.51 ± 0.03
100% NPs	97.49 ± 0.05	39.00 ± 0.02
150% NPs	98.70 ± 5.10	35.89 ± 1.86
200% NPs	99.11 ± 0.06	26.43 ± 0.02

SN38 7-Ethyl-10-hydroxycamptothecin, *SD* standard deviation, *NPs* nanoparticles

Table S2 Characterization of SN38 prodrug NPs (0.4 mg/ml) (n = 3, mean ± SD)

**Formulations**	**Size (nm)**	**PDI**	**Zeta potential (mv)**	**Encapsulation efficiency (%)**	**Drug loading (w/w, %)**
5% NPs	84.12 ± 1.44	0.17 ± 0.05	-19.37 ± 0.05	96.42 ± 1.81	73.46 ± 1.38
20% NPs	104.50 ± 2.19	0.09 ± 0.07	-18.93 ± 0.82	98.23 ± 1.68	65.49 ± 1.12
40% NPs	119.20 ± 0.91	0.05 ± 0.04	-19.53 ± 0.34	96.39 ± 0.80	55.08 ± 0.45
60% NPs	120.40 ± 2.14	0.12 ± 0.10	-21.00 ± 0.78	98.44 ± 1.38	49.22 ± 0.69
80% NPs	121.30 ± 3.82	0.06 ± 0.05	-19.37 ± 0.25	98.17 ± 2.52	43.63 ± 1.12
100% NPs	140.30 ± 5.45	0.16 ± 0.13	-22.53 ± 1.11	97.36 ± 1.19	38.94 ± 0.48
150% NPs	145.60 ± 5.63	0.15 ± 0.07	-22.17 ± 1.47	98.02 ± 0.65	35.64 ± 0.24
200% NPs	889.90 ± 73.11	1.00 ± 0.00	-	-	-

SN38 7-Ethyl-10-hydroxycamptothecin, *SD* standard deviation, *NPs* nanoparticles, *PDI* polydispersity index, "-" indicates due to the inability to form 200% NPs at 0.4 mg/ml, this study did not investigate further

Table S3 IC_{50} of SN38 sol, CPT-11, and SN38 prodrug NPs (nmol/L, *n* = 3, mean ± SD)

Formulations	CT26 cells	4T1 cells
SN38 sol	122.57 ± 13.80	24.88 ± 1.78
CPT-11	21,006.30 ± 1131.61	5746.33 ± 493.40
5% NPs	292.80 ± 11.95	76.94 ± 1.62
20% NPs	235.87 ± 13.52	48.97 ± 4.58
40% NPs	249.30 ± 2.76	50.97 ± 1.26
60% NPs	244.77 ± 6.41	53.89 ± 2.62
80% NPs	178.60 ± 5.31	41.03 ± 0.81
100% NPs	243.53 ± 8.21	51.53 ± 2.43
150% NPs	285.20 ± 5.77	64.66 ± 1.41
200% NPs	513.33 ± 17.44	84.94 ± 2.62

SN38 7-Ethyl-10-hydroxycamptothecin, *CPT-11* irinotecan, *SD* standard deviation, *SN38* sol SN38 solution, *NPs* nanoparticles

Table S4 Pharmacokinetic profiles of SN38 sol, CPT-11, and SN38 prodrug NPs (*n* = 4, mean ± SD)

Formulations	$AUC_{0\text{-}24\ h}$[a] (nmol·h/ml)	$T_{1/2}$[b] (h)	CLz[c] [L/(h·kg)]	Leakage rate of SN38 (%)
SN38 sol	0.08 ± 0.04	0.19 ± 0.05	306.21 ± 157.13	
CPT-11				
Prodrug	1.98 ± 0.55	0.71 ± 0.29	9.49 ± 2.67	
SN38	2.65 ± 0.30	1.44 ± 0.30	6.54 ± 0.34	
20% NPs				
Prodrug	2.33 ± 0.31	0.12 ± 0.08	8.59 ± 1.32	47.71 ± 5.01
SN38	2.33 ± 0.45	0.36 ± 0.12	9.47 ± 1.24	
80% NPs				
Prodrug	4.56 ± 0.47	0.14 ± 0.05	4.37 ± 0.40	46.14 ± 5.30
SN38	3.92 ± 0.62	0.56 ± 0.12	5.19 ± 0.75	
150% NPs				
Prodrug	58.96 ± 6.10	18.43 ± 4.41	0.21 ± 0.04	1.91 ± 0.48
SN38	1.13 ± 0.28	0.84 ± 0.24	17.10 ± 4.24	

[a]Area under the plasma concentration-time curve. [b]Half-life. [c]Plasma clearance. *SN38* 7-Ethyl-10-hydroxycamptothecin, *CPT-11* irinotecan, *SD* standard deviation, *SN38* sol SN38 solution, *NPs* nanoparticles
